# Structure and composition of a canopy-beetle community (Coleoptera) in a Neotropical lowland rainforest in southern Venezuela

**DOI:** 10.1098/rsos.240478

**Published:** 2024-07-17

**Authors:** Susan Kirmse

**Affiliations:** ^1^ Department of Ecology and Evolutionary Biology, University of California Santa Cruz (UCSC), Santa Cruz, CA, USA

**Keywords:** biodiversity, Amazonas, community structure, taxonomic composition, canopy crane

## Abstract

Species richness, community structure and taxonomic composition are important characteristics of biodiversity. Beetle communities show distinct diversity patterns according to habitat attributes. Tropical rainforest canopies, which are well known for their richness in Coleoptera, represent such a conspicuous life zone. Here, I describe a canopy-inhabiting beetle community associated with 23 tree species in a Neotropical lowland rainforest. Adult beetles were sampled manually and in aerial traps using a large tower crane for a cumulative year. The sample revealed 6738 adult beetles, which were assigned to 862 (morpho-)species in 45 families. The most species-rich beetle families were Curculionidae (*n* = 246), Chrysomelidae (*n* = 121) and Cerambycidae (*n* = 89). The most abundant families were Curculionidae (*n* = 2746) and Chrysomelidae (*n* = 1409). Dominant beetle families were found in most assemblages. The beetle community consisted of 400 singletons (46.4%). A similar proportion was evident for assemblages of single tree species. I found that 74.5% of all beetle species were restricted in their occurrence on host trees to the phenological season and time of the day. This daily and seasonal migration causes patterns similar to mass effects and therefore accounts for the high proportion of singletons.

## 1. Introduction

Rainforests comprise approximately half of all species [1]. Therefore, they are important diversity hotspots and in the focus of diversity research. Within tropical rainforests, tree canopies often harbour more species-rich communities than understorey habitats [2,3]. Particularly, beetles belong beside Hymenoptera and Diptera to the most abundant insect orders in this life zone [4–6]. Thus, the canopy of tropical rainforests provides crucial information regarding the function of forest ecosystems [7–9]. To understand ecological processes, it is important to study pristine forests [10–12], as there is growing evidence that disturbed forests differ fundamentally from primary forests with respect to species diversity and system services [13]. The rainforests of the Amazonas are of particular interest, as the New World tropical forests are more biodiverse than those in the Old World, and the forests of the Amazon Basin harbour more species than forests in Central America [14].

Tree canopies are exposed habitats that are characterized by harsh environmental conditions. They receive high levels of solar radiation, causing high fluctuations in relative humidity, air temperature and wind velocities compared with the understorey [15,16]. Therefore, arboreal animals must be at best tolerant to these conditions [17,18]. For that reason, the structure and composition of canopy communities should reflect the specific conditions of this life zone.

Tropical rainforest canopies not only harbour an overall high diversity but also many species represented only by a single specimen within assemblages [19–22]. Even with long-term sampling regimes, species accumulation curves of several tropical insects might not attain asymptotes [23–28], although, in general, ecological communities are assembled by a few abundant and many rare species independent of the habitat [29,30]. There are several forms of rarity that might be influenced by geographic range, abundance and habitat specialization [31,32]. However, little is known about the assembly of tropical arthropod communities [33].

On higher taxonomic level, the composition of beetle communities in forest canopies seems to be rather regular [22,23,34–36]. Comparing the species richness of beetle families in canopy samples, there was a similar taxonomic group composition, for instance, in communities in Panama [23] and Borneo [24]. The high-rank correlation coefficient for the number of species in different families or subfamilies of beetles showed the similarity in the beetle fauna of trees in the two regions at this taxonomic level. In addition, Stork [8,37] showed that the trophic guild structure of canopy arthropods is very similar for trees in temperate and subtropical forests [38], and for trees in tropical rainforests [37]. There is also a general similarity in community structure between temperate and tropical forests [39]. However, owing to a clear stratification of forest-inhabiting insect species, species assemblages and community structure in the canopy layer are specific [39–41].

To date, no comprehensive data or long-term studies have been published on the canopy of any near-equatorial rainforest worldwide [42]. I will provide such data for a canopy-inhabiting beetle community collected from 23 canopy-tree species in the Amazonas, 3° north of the equator, sampled for a cumulative year. Moreover, most canopy community data are sampled by fogging and include beetles associated with trunks, branches, epiphytes and phytotelmata from nearly all forest layers [43–45]. In contrast, the Venezuelan canopy sample predominantly includes beetles collected from leaves, smaller twigs, flowers and fruits, and therefore represents tighter host-specific associations with tree crowns [46]. Previously, I analysed the methods used in this survey (Kirmse, in preparation) and compared species richness and host specialization with studies from Panama [47], New Guinea [48] and Australia [49]. My results are in concordance with these studies, and thus, are suitable for analysing the composition and structure of the canopy-beetle community from this pristine lowland rainforest.

## 2. Material and methods

### 2.1. Study site

The study site is located in the upper Orinoco region (Venezuela, state of Amazonas) (3°10′ N, 65°40′ W; 105 m a.s.l.). While the Surumoni crane plot is located in the Orinoco depression, the area is part of the very old Guiana Shield bordered to the north by the Duida–Marahuaca massif [50]. A canopy crane was installed at the small black-water river Surumoni, a tributary of the large white-water river Orinoco. The tower crane was 42 m high and ran on 120 m long rails. An area of about 1.4 ha was accessible with the crane’s 40 m long swing. A gondola carrying scientists and their equipment enabled movement between the tree crowns.

The annual precipitation in the study area is approximately 3100 mm [51]. Precipitation strongly peaks from May to July and weakly in September and October. Year-to-year fluctuations of nearly 500 mm are common. 1997 was with only 2399 mm unusually dry owing to a strong El Niño event at the end of this year. The average annual temperature is *ca* 26°C, with slight variations between the coolest month (25°C) and the warmest month (26.5°C). Maximum temperatures during the day may reach 30.5°C and drop to only 20–21°C during the night.

The Surumoni area belongs to the Japura/Negro moist forest ecoregion [52] or Imerí province [53], which extends from Brazil to southern Venezuela, Colombia and Peru. The vegetation in this remote part of lowland moist forest in the northern Amazon Basin is classified as terra firme [54]. The upper canopy of the forest in the area usually ranges from 25 to 27 m in height. Only a few emergent trees rose to a height of 35 m. Crown closure was irregular and interrupted by light gaps. The Surumoni canopy-crane plot contained the average tree species richness of the area. There were more than 800 trees at least 10 cm in diameter at breast height (DBH) belonging to 141 tree species. Frequent species in the tree fraction with a DBH of at least 10 cm were *Dialium guianense* (Aubl.) Sandwith (Fabaceae), *Goupia glabra* Aubl. (Goupiaceae), *Ocotea* aff. *amazonica* (Meisn.) Mez (Lauraceae), *Oenocarpus bacaba* Mart. (Arecaceae) and *Ruizterania trichanthera* (Spruce ex Warm.) Marc.-Berti (Vochysiaceae) [55]. Epiphytes and hemiepiphytes were rare compared with other moist forests, comprising 53 species, with Araceae reaching the highest abundance [56]. These epiphytes were only found on 138 trees in the crane plot because of the absence of a pronounced canopy substrate. However, epiphyte composition resembles that of other lowland forests in this region.

### 2.2. Field studies

The general beetle survey was conducted as part of the interdisciplinary research project ‘Towards an understanding of the structure and function of a Neotropical rainforest ecosystem with special reference to its canopy’ organized by the Austrian Academy of Science. The cooperation with the Venezuelan government ended formally in 2000, as the political situation changed; therefore, the Surumoni crane project was closed. The beetle survey aimed to evaluate the association between adult beetles and their host trees over the course of a year. The field study was conducted between 1997 and 1999. Observations and collections of beetles comprised the following periods: September–November 1997; May–August and December 1998; January–April 1999, thus enabling data collection for a full cumulative year. Additional aerial trap collection targeting a single tree species was performed in October 1999.

The beetle survey includes 23 tree species representing 13 plant families in the upper (approximately 25−30 m height) and middle (approximately 18−25 m height) canopy: Annonaceae: *Guatteria schomburgkiana* Mart. and *Xylopia amazonica* R. E. Fr.; Apocynaceae: *Couma utilis* (Mart.) Müll. Arg.; Arecaceae: *Euterpe precatoria* Mart. and *O. bacaba*; Chrysobalanaceae: *Hymenopus heteromorphus* (Benth.) Sothers and Prance, *Licania hebantha* Mart. ex Hook. f. and *Moquilea subarachnophylla* (Cuatrec.) Sothers and Prance; Fabaceae: *Balizia pedicellaris* (DC.) Barneby and J. W. Grimes, *D. guianense*, *Senna* cf. *silvestris* (Vell.) H. S. Irwin and Barneby and *Tachigali guianensis* (Benth.) Zarucchi and Herend.; Goupiaceae: *G. glabra*; Lauraceae: *Ocotea* aff. *amazonica* and *Rhodostemonodaphne grandis* (Mez) Rohwer; Metteniusaceae: *Emmotum acuminatum* (Benth.) Miers; Urticaceae: *Pourouma melinonii* Benoist; Picrodendraceae: *Podocalyx loranthoides* Klotzsch; Rubiaceae: *Ferdinandusa* cf. *elliptica* (Pohl) Pohl; Sapindaceae: *Matayba guianensis* Aubl.; Vochysiaceae: *Qualea paraensis* Ducke, *R. trichanthera* and *Vochysia vismiifolia* Spruce ex Warm.

The trees selected for the canopy-beetle survey were either completely free from epiphytes and lianas or bore only small to minimize errors in beetle–host associations. Parts of the tree crowns sampled comprised leaves, small twigs, flowers and fruits. The selected trees were regularly searched for Coleoptera during the day and night. One individual of every tree species was controlled once per week for adult beetles. Four tree species were monitored every second day. This also applies to other tree species during distinct phenological seasons, such as flowering or leaf flush. A detailed description of the study design has been published in Kirmse (in preparation). Depending on the number of crown-associated beetles, the sampling time per tree crown was approximately 30 min from one gondola position.

The observed beetles were captured by net, hand or branch and foliage beating. However, these collection methods were not structured to provide quantitative data. To obtain standardized semi-quantitative sampling results, aerial traps were used to collect flying beetles [57]. These flight interception traps consisted of two clear acrylic panels fixed in a cross, each with a length of 30 cm and height of 25 cm. A plastic tube, ending in a container, was placed beneath the cross. The container was filled with water mixed with surface-tension-diminishing detergent. The insects trapped in the containers were removed every second day. In addition to hand and trap collection, some adult beetles were collected that were attracted to a spotlight used to enable observations at night.

### 2.3. Beetle processing and analyses

Collected beetles were stored in 70% ethanol. The beetles were assigned to morphospecies (hereafter, species), and the families were assigned according to family keys [58–60]. The beetles were later, in part, pinned and identified by experts. Buprestidae by C. L. Bellamy; Cantharidae and Lycidae by A. S. Ramsdale; Cerambycidae by U. R. Martins; Chrysomelidae by L. Chamorro, S. M. Clark, W. Flowers, D. Furth, L. N. Medvedev and D. Sassi; Cleridae by W. Opitz; Brentidae and Curculionidae by S. A. Vanin; Elateridae by P. J. Johnson; Lampyridae by V. R. Viviani; Mordellidae by J. A. Jackman; Scarabaeidae by B. C. Ratcliffe; and Tenebrionidae by M. Lillig. In addition, the author identified some beetle taxa based on available identification literature. Family group names follow Bouchard *et al*. [61]. Voucher specimens of collected beetles were deposited in the Museo del Instituto de Zoología Agrícola ‘Francisco Fernandez Yepez’, Maracay, Venezuela.

Every individual adult beetle collected from the 23 target tree species was included in the dataset (table 1). To analyse the occurrence of the beetle families in the 23 assemblages, the assemblages were grouped according to their species richness (Kirmse in preparation). The top five assemblages contain at least 100 beetle species and 15 assemblages at least 30 beetle species.

**Table 1.** Adult beetles (Coleoptera) collected (hand and trap samples combined) from 23 canopy-tree species (45 individuals) of a lowland tropical rainforest in Venezuela for a cumulative year between 1997 and 1999. The number of family occurrence is indicated in the five (at least 100 spp.) most species-rich assemblages, in the assemblages with at least 30 collected species (*n* = 15), and in all 23 assemblages.

family	no. spp.	no. indiv.	no. singl.	% singl.	no. spp. *n* ≥ **10**	% spp. *n* ≥ **10**	Ø indiv. / sp.	# described spp.	ref.	5 assembl. (≥100 spp.)	15 assembl. (≥30 spp.)	all 23 assembl.
Curculionidae Latreille	246	2746	107	43.5%	40	16.2%	11.16	53 000	[62]	5	15	23
Chrysomelidae Latreille	121	1409	44	36.4%	33	27.3%	11.6	47 600	[63]	5	14	22
Cerambycidae Latreille	89	274	49	55.1%	6	6.7%	3.08	38 300	[64]	5	12	16
Carabidae Latreille	75	202	40	53.3%	2	2.7%	2.7	40 000	[65]	5	14	19
Tenebrionidae Latreille	37	84	22	59.5%	2	5.4%	2.27	30 000	[66]	5	12	16
Scarabaeidae Latreille	33	167	10	30.3%	4	12.1%	5.06	27 800	[67]	5	13	18
Nitidulidae Latreille	22	346	11	50%	3	13.6%	15.73	4500	[65]	4	9	11
Mordellidae Latreille	22	206	4	18.2%	5	22.7%	9.36	2300	[68]	3	7	10
Elateridae Leach	20	380	10	50%	4	20%	19	12 000	[69]	5	14	16
Buprestidae Leach	20	145	11	55%	4	20%	7.25	14 700	[65]	4	9	10
Staphylinidae Latreille	17	126	8	47.1%	3	17.6%	7.41	61 300	[70]	3	6	7
Brentidae Billberg	16	32	9	56.2%	0	0	2	4400	[71]	4	6	8
Cantharidae Imhoff	15	91	5	33.3%	2	13.3%	6.07	5700	[72]	5	13	15
Coccinellidae Mulsant	15	37	10	66.7%	0	0	2.47	6000	[65]	4	9	12
Dermestidae Latreille	10	89	1	10%	3	30%	8.9	1440	[73]	3	7	8
Scirtidae Fleming	9	17	5	55.6%	0	0	1.89	800	[65]	4	8	9
Cleridae Latreille	8	71	2	25%	2	25%	8.88	3550	[74]	4	10	12
Eucnemidae Eschscholtz	8	9	7	87.5%	0	0	1.13	1900	[75]	3	4	5
Lycidae Laporte	7	12	4	57.1%	0	0	1.71	4200	[76]	3	4	4
Histeridae Gyllenhal	6	8	4	66.7%	0	0	1.33	4300	[65]	4	6	6
Zopheridae Solier	5	74	0	0	3	60%	14.8	1700	[65]	4	10	14
Hybosoridae Erichson	5	73	2	40%	1	20%	14.6	570	[65]	4	8	12
Anthribidae Billberg	5	9	2	40%	0	0	1.8	3900	[65]	2	4	5
Melyridae Leach	5	8	3	60%	0	0	1.6	6000	[65]	3	3	4
Trogossitidae Latreille	5	5	5	100%	0	0	1	600	[65]	2	3	3
Ptilodactylidae Laporte	4	24	1	25%	1	25%	6	500	[65]	4	9	12
Lampyridae Rafinesque	4	19	3	75%	1	25%	4.75	2200	[65]	2	6	6
Attelabidae Billberg	4	7	3	75%	0	0	1.75	2500	[65]	0	4	4
Meloidae Gyllenhal	3	12	1	33.3%	0	0	4	3000	[65]	0	2	2
Oedemeridae Latreille	3	6	2	66.7%	0	0	2	500	[65]	2	4	4
Ptinidae Latreille	3	5	2	66.6%	0	0	1.7	2200	[65]	2	4	4
Phalacridae Leach	2	11	1	50%	1	50%	5.5	640	[65]	2	3	3
Leiodidae Fleming	2	8	1	50%	0	0	4	3700	[65]	2	4	4
Scraptiidae Gistel	2	6	1	50%	0	0	3	500	[65]	2	4	4
Corylophidae LeConte	2	5	1	50%	0	0	2.5	200	[65]	1	2	2
Nemonychidae Bedel	2	3	1	50%	0	0	1.5	75	[77]	0	1	1
Hydrophilidae Latreille	2	2	2	100%	0	0	1	3400	[65]	1	1	2
Endomychidae Leach	1	2	0	0	0	0	2	1800	[65]	1	1	1
Cryptophagidae Kirby	1	2	0	0	0	0	2	600	[65]	1	1	1
Erotylidae Latreille	1	1	1	100%	0	0	1	3500	[65]	1	1	1
Elmidae Curtis	1	1	1	100%	0	0	1	1500	[65]	1	1	1
Passalidae Leach	1	1	1	100%	0	0	1	930	[78]	0	1	1
Aderidae Csiki	1	1	1	100%	0	0	1	900	[62]	1	1	1
Chelonariidae Blanchard	1	1	1	100%	0	0	1	300	[79]	1	1	1
Cucujidae Latreille	1	1	1	100%	0	0	1	44	[80]	1	1	1
Total	862	6738	400		120							

For diversity analyses (table 3), the three most species-rich flower-visiting beetle assemblages collected from a single tree during one flowering season (*H. heteromorphus* [81], *M. guianensis* and *T. guianensis* [82]) were selected. For these three flower-visiting assemblages, I present the trap samples and, in addition, for two, the total sample combining hand and trap collections. I chose further two beetle assemblages visiting extrafloral nectaries (*L. hebantha* and *M. subarachnophylla* [83]) and four assemblages collected in all phenological seasons for the aspect of a year (*G. glabra*, *R. trichanthera*, *S.* cf. *silvestris* and *T. guianensis*). Because of the overall lower trap catches, only the total samples of the two extrafloral nectary and four year–aspect assemblages were analysed. For single rarefaction, I used the total samples of flower-visiting assemblages of *H. heteromorphus* and *T. guianensis* and the total assemblages of *G. glabra*, *R. trichanthera* and *S.* cf. *silvestris* (table 3). The flower-visiting assemblage of *M. guianensis* comprises the total sample of two tree individuals from three flowering seasons (260 species, 1456 individuals [81]). For sample rarefaction, all beetle species and individuals sampled from the 23 tree species over the course of the year were included.

### 2.4. Statistical analyses

To characterize the diversity of assemblages, Chao *et al*. [84] proposed an integrated approach based on Hill numbers [85], which comprises the three most common species diversity measures (species richness, Shannon and Simpson diversity). Diversity characteristics should include a sample completeness profile, asymptotic diversity estimates, non-asymptotic standardization via rarefaction and extrapolation and an evenness profile. PAST (v. 3.21 [86]) was used to calculate the indices and non-parametric correlations.

The following indices were computed to characterize the alpha diversity and dominance distribution of the assemblages (table 3): Shannon_H, Evenness_e^H/S, Simpson_1-D, Dominance_D and Berger–Parker. As alpha diversity measures relate the distribution of sampled individuals to the species, high values may be caused either by increasing species richness or by the uniform distribution of individuals among species. High values of the Shannon–Wiener index [87] indicate many taxa, each with few individuals. This index H is suitable for random assemblages of large communities with a known total species number [88]. The value is strongly influenced by the sample size and abundance of the middle common species. If the number of individuals increases, but the structure and number of species remain at the same level than there is no influence on the index. The evenness is derived from the Shannon–Wiener index, as the index alone makes not clear, if its value is caused by many species or by the uniform distribution of individuals among the species. Evenness indicates the relationship between the diversity index and the theoretical maximal index value under the highest possible uniform distribution of individuals to species [89]. A high evenness is expressed by a uniform distribution. The Simpson index D [90] shows the probability that two individuals of one assemblage belong to different species. This value is determined by the dominance of the most abundant species and does not count rare species with only one collected specimen. The Simpson index has always been regarded as a classical dissimilarity measure that is insensitive to differences in richness between two communities [91,92]. The Simpson index 1-D measures the evenness of the community. The dominance Simpson index ranges from 0 if all species are equally present to 1 if only one species completely dominates the community. The Berger–Parker index [93] does not depend on species richness. It is based on the proportional importance of the most abundant species. The value is lower when the dominance pyramid is more uniform.

**Figure 1.**
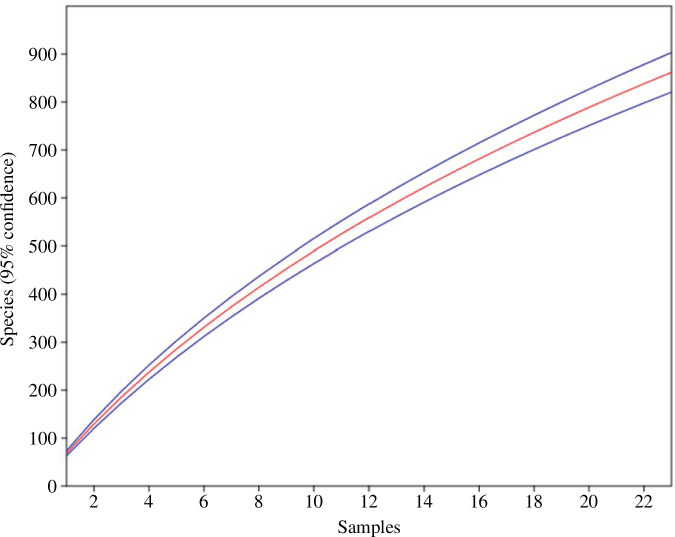
Sample rarefaction Mao’s Tau of beetle assemblages collected from 23 canopy-tree species (45 individuals) of a lowland tropical rainforest in Venezuela between 1997 and 1999.

**Figure 2.**
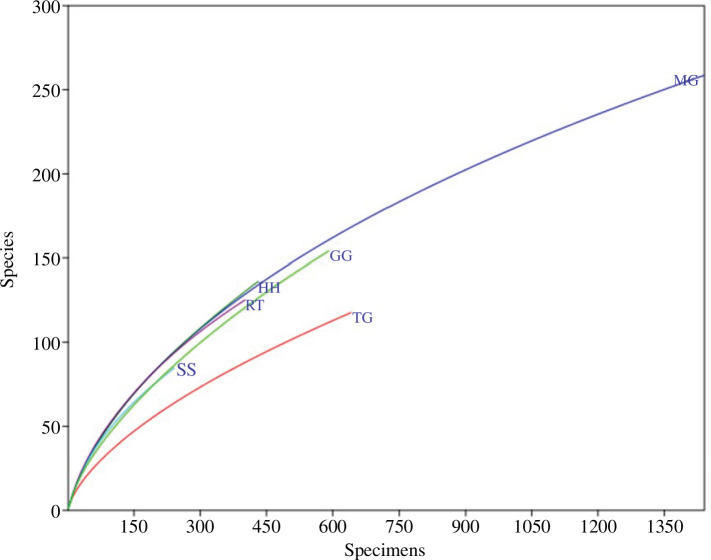
Single rarefaction of most species-rich beetle assemblages from canopy-tree species of a lowland tropical rainforest in Venezuela between 1997 and 1999. Flower-visiting assemblages: (HH) *H. heteromorphus*, (MG) *M. guianensis*, (TG) *T. guianensis* year–aspect assemblages: (GG) *G. glabra*, (RT) *R. trichanthera*, (SS) *S*. cf. *silvestris*

Rarefaction was used to estimate the species richness of the assemblage independent of the sampling effort [94]. Rarefaction places the number of expected species in relation to the number of individuals on the x-axis (figures 1 and 2). Using the same sampling technique, the result is a characteristic species accumulation curve with increasing sample size. A high degree of evenness slows down the increase in the curve. Rare species have the greatest influence on species-rich communities, expressed by a steep increase in the curve indicating a species-rich community. Furthermore, I computed sample rarefaction or species accumulation curve with a 95% confidence interval based on presence–absence data for the total assemblage of 23 tree species. PAST uses the expected species richness function Mao’s Tau with species richness estimated as function of the number of samples.

Finally, I used the non-parametric Spearman’s correlation coefficient to analyse whether the sample size of different beetle families is correlated with the number of rare and abundant species, the number of known species and their representation in the single assemblages (table 2). Spearman’s rank-order correlation coefficient is the linear correlation coefficient (Pearson’s *r*) of the ranks.

**Table 2.** Spearman’s correlation coefficients of species number per family in relation to several parameters for the beetle assemblages collected from 23 canopy-tree species (45 individuals) of a lowland tropical rainforest in Venezuela between 1997 and 1999. *r_S_* values in lower, and *p* values upper matrix.

	# species	# individuals	# singletons	# species *n* ≥ 10 individuals	Ø individuals per species	# described species	occurrence in assembl. with ≥100 spp.	occurrence in assembl. with ≥30 spp.	occurrence in all 23 assembl.
# species		<0.0001	<0.0001	<0.0001	<0.0001	<0.0001	<0.0001	<0.0001	<0.0001
# individuals	0.92901		<0.0001	<0.0001	<0.0001	<0.0001	<0.0001	<0.0001	<0.0001
# singletons	0.8844	0.72186		<0.0001	0.0416	<0.0001	<0.0001	<0.0001	<0.0001
# species *n* ≥ 10 individuals	0.74426	0.86903	0.52748		<0.0001	<0.0001	<0.0001	<0.0001	<0.0001
Ø individuals per species	0.60922	0.84362	0.305	0.8142		0.01349	<0.0001	<0.0001	<0.0001
# described spp.	0.75062	0.6767	0.76276	0.53638	0.36572		<0.0001	<0.0001	<0.0001
occurrence in assembl. with ≥100 spp.	0.87532	0.85596	0.74386	0.72097	0.619	0.64571		<0.0001	<0.0001
occurrence in assembl. with ≥30 spp.	0.89542	0.92706	0.72082	0.79464	0.75237	0.63357	0.9341		<0.0001
occurrence in all 23 assembl.	0.90823	0.92831	0.73404	0.7885	0.73213	0.64706	0.94142	0.9899	

## 3. Results

### 3.1. Richness of the canopy-beetle community

In total, the survey revealed 6738 adult beetles on 23 canopy-tree species over the course of a year (table 1). These adults were assigned to 862 beetle (morpho-)species from 45 families. Four hundred species (46.4%) were represented by a single individual. In contrast, only 120 species (13.9%) were collected with at least 10 individuals. On average, 7.82 individuals were collected per species. The sample rarefaction curve on basis of Mao’s Tau shows that there is no slowdown of the steep increase (figure 1).

### 3.2. Composition of the canopy-beetle community

In the entire canopy community, Curculionidae are represented by most species (*n* = 246), followed by Chrysomelidae (*n* = 121), Cerambycidae (*n* = 89) and Carabidae (*n* = 75) (table 1). Most individuals were collected from Curculionidae (*n* = 2746) and Chrysomelidae (*n* = 1409). Curculionidae were sampled from every tree species and Chrysomelidae from 22 of the 23 tree species.

Other species-rich beetle families with at least 10 collected species are Tenebrionidae, Scarabaeidae, Nitidulidae, Mordellidae, Elateridae, Buprestidae, Staphylinidae, Brentidae, Cantharidae, Coccinellidae and Dermestidae. Species of these families were found on at least seven tree species and in three of the five most species-rich assemblages. Cleridae, Zopheridae, Hybosoridae and Ptilodactylidae were represented in four of the five most species-rich assemblages and were collected from 12 to 14 tree species, although they comprise fewer than 10 species.

The proportion of singletons per family ranges from 0 to 100% and considering only the 15 beetle families with at least 10 species from 10 to 66.7%. Mordellidae (18.2%) and Dermestidae (10%) have the lowest proportions and were sampled with on average 9.36 and 8.9 individuals per species. In contrast, Cerambycidae, Carabidae and Tenebrionidae comprise more than 50% of singletons. Their average numbers of individuals per species are only 3.08, 2.7 and 2.27, respectively.

The proportion of species with at least 10 collected individuals per family ranges from 0 to 60%. Among the 15 beetle families with at least 10 species, Coccinellidae and Brentidae have no such abundant species, whereas the proportion of Chrysomelidae, Mordellidae, Elateridae, Buprestidae and Dermestidae is between 20 and 30%. Coccinellidae and Brentidae were sampled on average with 2.47 and 2 individuals per species, respectively, but the other five families on average with at least 7.25 individuals per species.

### 3.3. Correlations of sample characteristics

The number of sampled species per beetle family is correlated with the number of individuals, the proportion of singletons and species collected with at least 10 individuals, as well as with the average number of individuals per species. The Spearman’s correlation coefficient shows a significant positive correlation for each of these parameters (table 2). There is also a significant correlation between the number of species per family and the occurrence of beetle families in the top five, the 15 most species-rich and in all 23 assemblages.

There is a significant tendency for species-rich beetle families to be found with commonly most species (*r_S_* = 0.75062) and individuals (*r_S_* = 0.6767) on the 23 target tree species (table 2). There are three notable exceptions to this trend. Staphylinidae currently comprise the most described species, but rank 11th according to their species richness on the 23 canopy-tree species (table 1). Nitidulidae and Mordellidae were abundant and represented with 22 species each in the cumulative assemblage, but the number of described species is with 4500 and 2300 species, respectively, clearly lower compared with the next three most species-rich beetle families (table 1).

### 3.4. Combining three sampling methods

Several species of Carabidae, Chrysomelidae, Elateridae and Scarabaeidae were attracted to the spotlight used to enable work at night. I add here the number of specimens sampled at light to the number of specimens collected from the 23 canopy-tree species. In Carabidae, 71 individuals belonging to 12 species were added. In Elateridae, I added 19 individuals from six species. In Scarabaeidae, I added a total of 62 individuals belonging to 13 species, and in Chrysomelidae, I added 320 specimens from 22 different species. As a result, the proportion of singletons decreases in Carabidae from 40 (53.3%) to 35 species (46.7%), in Elateridae from 10 (50%) to 8 species (40%), in Scarabaeidae from 10 (30.3%) to 6 species (18.2%), and in Chrysomelidae from 44 (36.4%) to 39 species (32.2%).

### 3.5. Structure of species-rich assemblages

Single rarefaction curves of the total samples (hand + trap collections) of the six most species-rich beetle assemblages show a steep increase (figure 2). They do not attain an asymptote because of the high proportion of singletons and rare species. This applies to predominantly flower-visiting communities collected on *H. heteromorphus*, *M. guianensis* and *T. guianensis* as well as for beetle assemblages collected during the aspect of a year on *G. glabra*, *R. trichanthera* and *S.* cf. *silvestris* covering different phenological seasons.

In every one of the selected 11 assemblages, the proportion of singletons ranges from 52 to 71% (table 3). This applies to beetle communities collected during a particular phenological season (flowering and leaf flushing with extrafloral nectaries) and beetle communities sampled over the course of a year. Combining trap collections with intensive hand collection, such as in *M. guianensis* and *H. heteromorphus*, the proportion of singletons decreases only slightly. However, there is no reduction in the number of singletons in *T. guianensis* comparing the trap sample during the flowering season with the total sample throughout the cumulative year comprising trap and hand collection. Up to 10% of the sampled species were represented by at least 10 individuals in the 11 selected assemblages. The extrafloral nectary-bearing trees *L. hebantha* and *M. subarachnophylla* do not comprise one species with at least 10 individuals. Instead, with on average 1.6 and 1.5 individuals per species, these assemblages were frequented by relatively few individuals. In contrast, the maximum abundance on flowering *M. guianensis* and *T. guianensis* is 127, with an average of 5.9 and 4.9 individuals per species, respectively.

**Table 3.** Comparison of species-rich adult beetle assemblages either collected with traps or hand and trap samples combined from eight canopy-tree species of a lowland tropical rainforest in Venezuela between 1997 and 1999.

	M.g. #446 1997	M.g. #446 1997	H.h.	H.h.	T.g.	T.g. total sample	L.h.	M.s.	S.s.	R.t.	G.g.
sample	trap	hand + trap	trap	hand + trap	trap	hand + trap	hand + trap	hand + trap	hand + trap	hand + trap	hand + trap
sampling period	flowering	flowering	flowering	flowering	flowering	year-round	leaf flush (EFN)	leaf flush (EFN)	year-round	year-round	year-round
# species	114	183	63	138	105	169	33	44	88	128	156
# individuals	535	905	159	440	622	794	53	65	257	418	603
# singletons	60	95	45	87	63	102	22	31	51	69	96
singletons %	52.6	51.9	71.4	63.0	60.0	60.4	66.7	70.4	58.0	53.9	61.5
# species *n* ≥ 10	11	19	2	7	9	14	0	0	6	11	14
species *n* ≥ 10%	9.6	10.4	3.2	5.1	8.6	8.3	0.0	0.0	6.8	8.6	9.0
individuals per species	4.7	4.9	2.5	3.2	5.9	4.5	1.6	1.5	2.9	3.3	3.9
max abundance	127	127	37	52	127	127	6	3	24	27	65
Berger–Parker	0.2378	0.1419	0.2327	0.1182	0.2042	0.161	0.1132	0.04615	0.09339	0.06444	0.1078
Simpson_1-D	0.9194	0.9547	0.9095	0.9657	0.9094	0.9416	0.9555	0.9709	0.9681	0.9747	0.9618
Dominance_D	0.08059	0.04528	0.09054	0.03434	0.09059	0.05837	0.0445	0.02911	0.03187	0.02533	0.03818
Shannon_H	3.537	4.076	3.292	4.128	3.225	3.832	3.319	3.662	3.932	4.207	4.023
Evenness_e^H/S	0.2989	0.3236	0.4267	0.4498	0.2418	0.273	0.8376	0.8851	0.5798	0.5244	0.358

EFN, active extrafloral nectaries; G.g., *Goupia glabra*; H.h., *Hymenopus heteromorphus*; L.h., *Licania hebantha*; M.g., *Matayba guianensis*; M.s., *Moquilea subarachnophylla*; R.t., *Ruizterania trichanthera*; S.s., *Senna cf. silvestris*; T.g., *Tachigali guianensis*.

The Simpson index is greater than 0.9 for all 11 assemblages, while the Shannon diversity is generally higher for the assemblages representing the aspect of a year and for the flower-visiting assemblages with combined trap and hand samples. The mean value of the Shannon index for the entire assemblage of 23 tree species is 5.26 higher compared with the single assemblages. The dominance Simpson index shows approximately two times higher values for the trap samples of the flowering trees in comparison with all other assemblages, indicating a more unequal distribution of the species. Similarly, the Berger–Parker index indicates a more heterogenic dominance pyramid for these trap samples. In contrast, the evenness values indicate a more uniform abundance distribution for both *L. hebantha* and *M. subarachnophylla* in comparison with the other assemblages.

## 4. Discussion

### 4.1. Sample completeness

According to a general analysis of the sampling methods (Kirmse in preparation), the beetle fauna of the selected 23 tree species was well collected during this survey. Host specificity with 23.7 exclusive species per tree species (6.3 beetle species excluding singletons) and a total of 68.3 beetle species associated with one canopy-tree species are in line with studies from other rainforest canopies (Kirmse in preparation). In addition, general beetle species richness, the high percentage of singletons, and the dominance of the herbivorous beetle families Chrysomelidae and Curculionidae are in concordance with other studies, thus validating the suitability of the data for analysing the composition and structure of the canopy-beetle community from this pristine lowland rainforest. However, sampling was restricted to a very small area of the tree crowns, resulting in a low number of individuals. Observations and collections within the canopy plot covered 23 tree species from 13 plant families with only a small proportion of potential host plants. Within the crane plot, there were 141 tree species and approximately 800 tree specimens with DBH at least 10 cm alone. Moreover, there were lianas and epiphytes. In total, 322 vascular plants from 78 families were recorded in the crane plot, which probably amounted to 350−400 plant species [55]. In conclusion, although I collected only a small proportion of the local beetle community, community structure and composition might be representative. A total of 862 beetle species from 45 families were collected from 23 canopy-tree species during the year of study. This study is comparable to a 1 year sample of canopy beetles collected from 23 canopy plants in an Australian rainforest. Wardhaugh [95] recorded 41 beetle families on flowers, and mature and new leaves. Although family systematics have been handled differently among studies, insecticide fogging methods often reveal more families associated with tropical rainforest trees. Davies *et al*. [36], for instance, fogged 65 beetle families in a montane Neotropical rainforest, Márquez *et al*. [96] fogged 52 families in a cloud forest in Mexico and Erwin and Scott [23] fogged 57 beetle families from a single tree species in Panama.

Regardless of the sampling of 23 tree species during the night and day for a cumulative year, neither the species accumulation curves of single tree species assemblages nor for the entire assemblage attained any asymptote (figures 1 and 2). This is a typical feature of many tropical communities and indicates that the samples contain only a part of the local community and potential species richness. In contrast, an asymptote of rarefaction or almost complete sampling can be reached, for instance, in temperate rainforests, where diversity is much lower. Arias *et al*. [97], for instance, collected 485 beetle morphospecies with 8−86 species and 168−294 individuals per tree from 29 trees of three different species in Chile. Species represented by one or two individuals amounted to only 0.5 and 0.3%, respectively. As a result, the total predicted number of species using Chao for the combined samples was 601. Sampling a beetle community more completely in complex tropical rainforests requires more intensive sampling. Erwin *et al*. [3] conducted such a mega-study in Western Amazonia. They fogged over 500 000 beetle specimens. For the 35 Otidocephalini species (Curculionidae), a combination of 300 samples was sufficient to achieve an almost complete sampling. The 462 species of Carabidae required an analysis of 1200 fogging events to come close to an asymptote of the species accumulation curve. Basset and Novotny [98] suggested that the commonly high aggregate distribution on a single host tree species is responsible for the accumulation curves not attaining an asymptote. However, owing to practical limitations, it is virtually impossible to detect all species, particularly in hyperdiverse assemblages with many rare species [99–102].

### 4.2. Taxonomic composition

Although the sampling method (e.g. fogging) may influence the record of families, the taxonomic composition of the canopy-beetle community resembles that of other tropical rainforests. In concordance with the Venezuelan sample, the herbivorous families Curculionidae and Chrysomelidae were collected in high abundance [4,36,95,103]. Within the central Amazonian forests, herbivorous Curculionidae predominated in all forest types, whereas Chrysomelidae ranked second [104]. In a montane Neotropical rainforest, Davies *et al.* [36] fogged predominantly many species of Curculionidae (*n* = 100), Staphylinidae (*n* = 90), Coccinellidae (*n* = 72), anobiine Ptinidae (*n* = 72) and bruchine Chrysomelidae (*n* = 60). In a tropical wet forest in western Kenya, Staphylinidae were the most species-rich group, followed by Chrysomelidae and Curculionidae [105]. Wagner [106] reported a similar family composition in Uganda, with most species collected from Staphylinidae, followed by Curculionidae and Chrysomelidae. Hammond *et al.* [21] found that Anthribidae, Cerambycidae and Mordellidae are occasionally dominant in arboreal communities in Sulawesi. Aderidae, Anthribidae, Chrysomelidae, Corylophidae, Curculionidae and Staphylinidae were species-rich families of Fagaceae in New Guinea [107]. Along with Curculionidae and Chrysomelidae, Coccinellidae, Nitidulidae and Phalacridae were the most species-rich families in the Australian rainforest [95]. On *Vochysia divergens* Pohl in the Pantanal of Brazil, Curculionidae were the most species-rich family, followed by Chrysomelidae and Staphylinidae [108]. On *Theobroma grandiflorum* (Willd. ex Spreng.) Schum. (Sterculiaceae) in the Brazilian Amazon, Graças *et al*. [109] sampled, combining fogging and flight interception trapping, predominantly Chrysomelidae (25%), Curculionidae (18%), Staphylinidae (18%) and Coccinellidae (12%). Five species of ceratocanthine Hybosoridae were represented in the Venezuelan canopy sample and in an Afrotropical rainforest [110].

In summary, lowland tropical rainforest tree communities are often dominated by a relatively small number of highly species-rich families [111]. Carabidae, Cerambycidae, Chrysomelidae, Curculionidae and Scarabaeidae are cosmopolitan and represent five of mega-diverse coleopteran families [112]. These highly species-rich families were often abundant within tree crowns (tables 1 and 2). This correlation has also been reported in other studies (e.g. [113]). Reviewed by Basset [114], Curculionidae, Chrysomelidae and Staphylinidae appear to be the most speciose taxa in tropical rainforest canopies. Most common families present in canopy samples include furthermore Anthribidae, Carabidae, Cleridae, Coccinellidae, Corylophidae, Latridiidae, Mordellidae, Phalacridae and Tenebrionidae. Remarkably, I sampled relatively few Staphylinidae, although this beetle family seems to be a regular part of arboreal communities in other rainforests [21,36,103,107]. In the Brazilian Pantanal, Staphylinidae were found to be one of the dominant and species-rich beetle families (*n* = 102) associated with the palm *Attalea phalerata* Mart [115]. In the Venezuelan canopy plot, Staphylinidae were particularly associated with the inflorescences of the palm *O. bacaba*. Staphylinidae may be more specious and abundant at the ground level [116]. However, apart from their close association with moisture [117], I did not sample arboreal humus accumulations or fungi, which are commonly included in insecticide fogging. The same may apply to the absence of Latridiidae in Venezuelan samples. Instead, I collected seven species of Lycidae that are common in humid tropical forests [118].

Unfortunately, most studies of canopy beetles lack data on the phenology or specific conditions of trees, such as the occurrence of epiphytes, fungi or dead wood, which may support different beetle taxa. It has long been known that some beetle families are associated with flowering trees [81]. In the Venezuelan assemblage, this applies particularly to Cerambycidae and Dermestidae. In a lowland dipterocarp forest in Malaysia, Chrysomelidae and Curculionidae were the most abundant beetle families during a general flowering period [119]. Sakai *et al*. [120] found Chrysomelidae, followed by Curculionidae and Nitidulidae, to be the predominant families among visitors. Kato *et al*. [121] revealed that Chrysomelidae, Scarabaeidae, Dermestidae, Nitidulidae and Curculionidae are the most abundant families. Similarly, Curculionidae, Chrysomelidae and Cerambycidae have been found to be the dominant flower visitors to two Neotropical canopy trees in wet Panamanian lowland forests [122].

Nevertheless, abundant and species-rich canopy-beetle families were present in most assemblages, as indicated by the significant positive correlation in the tree–crown assemblages (table 2). This relationship is confirmed in a large study in the western Amazonian rainforest by Erwin *et al*. [3]. Carabidae were sampled with 462 morphospecies in 1200 samples studied, and occurred in 76% of the samples. Buprestidae occurred in 47% of 600 samples studied, and Cleridae occurred in 50% of 1200 samples. Similarly, the eight Venezuelan species of Cleridae occurred in 52% and Buprestidae in 43% of the assemblages, whereas Carabidae were found on 83% of all 23 tree species. Furthermore, species-rich beetle families occurred with most species on a tree. However, depending on the resources used, a few families fail to follow this trend. For instance, buprestid species richness ranked 10th in the Venezuelan canopy community, but according to the number of described species eighth, and they were collected only in 10 assemblages. This discrepancy may be attributed to more specific host associations. In contrast, Dermestidae comprise relatively few described species (31st rank), but rank 15th in species richness in the canopy assemblage. They were collected only from eight flowering trees indicating a strong association with this food resource in tree crowns.

### 4.3. Community structure

Most species are relatively rare in the vast majority of communities, particularly in species-rich tropical communities, including insect herbivores [25,98,123,124]. This pattern has been confirmed by many studies, mostly independent of the sampling technique, and applies, for instance, to boreal forest carabid communities [125] or Coleoptera in different forests in Amazonia [104]. This relationship is maintained even in Erwin *et al*.’s [3] large study in the western Amazonian rainforest. In 122 species of Cleridae, the three most common species accounted for 35.94% of the abundance, and in otidocephaline Curculionidae, the three most common species accounted for 44.9% of the abundance. Lucky *et al*. [126] collected in 900 canopy fogging samples over 3 years in Amazonia 318 carabid species comprising only 10 species in abundances greater than 50 individuals amounting to 31.9% of all individuals. In the Venezuelan canopy community, only 120 species (13.9%) were collected with at least 10 individuals. Yet, the proportion of species with at least 10 individuals was with maximal 10% lower in the single assemblages, indicating that increasing the number of investigated tree species will increase the overall abundances in the sample. The overall proportion of species with at least 10 individuals is very similar to that reported in other studies in tropical rainforest canopies. In Papua New Guinean forest canopies, Allison *et al*. [20] sampled 14.8% of the beetle species with at least 10 individuals, and Wagner [106] sampled 10.4% in Ugandan forest canopies. Fogging of the canopy of a cloud forest in Mexico revealed that only 17.3% of 325 morphospecies were collected with more than 10 individuals [96]. In the Pantanal of Brazil, 26.6% of the species were collected with at least 10 individuals on *V. divergens* [108] and 21.8% on the palm *A. phalerata* [127]. However, the percentage of species collected with at least 10 individuals ranges from 0 to 60% per family in the Venezuelan canopy community. Among the families represented by at least 10 species, Dermestidae with 30% and Chrysomelidae with 27.3% reach the highest proportion. This is because all species of Dermestidae and the majority of Chrysomelidae were sampled exclusively from flowering trees, where some species aggregated in high numbers.

Nevertheless, the abundance of beetles in the canopy assemblage was low, with the highest average number of individuals per species collected from flowering trees. MacArthur [128] pointed out that, in general, there is a negative relationship between species richness and dominance. Consequently, species-rich habitats, such as tropical rainforest canopies, have a high proportion of rare species [129,130]. However, the overall low abundances in the Venezuelan survey were largely because only a small part of the tree crowns were sampled and hand collections were not structured to obtain quantitative data. Among the beetle families, Elateridae were collected on average with 19 individuals per species, but this was owing to one abundant species. I found similarly high average numbers in Nitidulidae, with most species only collected from flowering trees. On average, both Curculionidae and Chrysomelidae, comprising many flower visitors, were collected with more than 11 individuals per species. In contrast, other families had only a few individuals per species (on average two–three individuals). Among the families collected with at least 10 species, this applies to Brentidae, Carabidae, Cerambycidae, Coccinellidae and Tenebrionidae. While Carabidae and Coccinellidae are predatory or omnivorous, the other families (subfamily Brentinae) comprise on average larger species than Chrysomelidae and Nitidulidae [131]. Large and predatory species have lower population densities and are, therefore, more likely to be sampled as singletons [132,133]. However, the overall abundance should be largely influenced by the quality and nutrient content of the food, as abundance in forest canopy arthropods is limited by resources and abiotic factors [134]. The Venezuelan crane plot is situated at a site with nutrient-poor soils [50,135]. Lamarre *et al*. [33] compared forest types in different regions of the Amazonas and found that most arthropod groups were more abundant in high-resource environments (terra firme forests on clay) than in low-resource habitats (white-sand forest). For instance, Chrysomelidae and Curculionidae supported 2.7 and 2.1 more individuals, respectively, in terra firme forests on clay than in white sand forests.

The proportion of singletons in the most species-rich assemblages of the 23 tree species ranged from 52 to 71%. Novotný and Basset [28] reported an average proportion of rare species of 45%, with no difference among plant species in their herbivore communities in New Guinea. The entire Venezuelan canopy-beetle community had a 46.4% lower value than the single assemblages. This result is in concordance with many other studies in tropical rainforests, although with differences depending on the sampling technique, sampled habitat, sampling effort, sampling period or taxa sampled. In the Pantanal of Brazil, the proportion of singletons was 39.8% on *V. divergens* [108] and 41.7% on the palm *A. phalerata* [127]. Erwin [136] found 50% of 3429 species in Peru only once. Didham *et al*. [137] reported a 45% proportion of singletons in an assemblage of 993 species from Brazil. Wagner’s [106] proportion of canopy-beetle singletons in Uganda was 41.6%. Monteith and Davies [138] recorded 40% singletons among 1514 species sampled in an Australian rainforest. Allison *et al*. [107] recorded 51% singletons among 633 species and 48% among 419 species [20] in New Guinea. Morse *et al*. [19] found that 58% of the beetles collected by canopy fogging were represented by only one individual in a tropical lowland rainforest in Brunei. Hammond *et al*. [21] sampled 47% of 1355 species only with a single individual in Sulawesi, while the fogging sample of Floren *et al*. [139] revealed a proportion of 51.9% in Java.

The proportion of singletons per family in the Venezuelan canopy community ranges from 0 to 100%. A high proportion of singletons can indicate that a community is undersampled [99,133,140]. However, I demonstrate that there are criteria that largely influence the proportion of singletons per family. Among the families with at least 10 collected species, the proportion was 10−66.7%. Dermestidae (10%) and Mordellidae (18.2%) had the lowest proportions of singletons. All species from these two families were collected exclusively from flowering trees. This indicates that sampling a family on their commonly used resources (their distinct habitat) enhances the probability of sampling complete species richness. In contrast, predominantly predatory Coccinellidae revealed a proportion of 66.7% singletons and Tenebrionidae comprising taxa of very different feeding guilds revealed a proportion of 59.5%. Supplementing the combination of hand and trap collection with specimens collected at light, the proportion of singletons decreased in Carabidae, Chrysomelidae, Elateridae and Scarabaeidae. In particular, Scarabaeidae [141,142], Carabidae, Chrysomelidae [143] and Elateridae are well represented in light trap collections [144]. The light samples will add specimens to the samples that occur on host trees that were not included in this survey. In Scarabaeidae, I could reach by combining these three sampling methods the low proportion of 18.2% singletons, indicating that I had good species coverage on the 23 tree species. Longino *et al*. [140] achieved a relatively complete inventory of spiders through a combination of non-quantitative specialist collection and quantitatively structured sampling. Using suitable sampling methods, a more complete inventory of single taxa can be obtained, even in species-rich tropical forests. Wolda [145] collected 703 species of Curculionidae in Panama using light traps, and reported a proportion of only 19% for singletons. Baited traps for dung beetles resulted in 73 species and only 10% singletons in Bolivia [146]. Comprehensive long-term sampling can also reduce the proportion of singletons in tropical rainforest canopy assemblages. While the Venezuelan carabid community consisted of 53.3% singletons, the proportion in the comprehensive study by Lucky *et al*. [126] was only 28.6%.

### 4.4. Reasons of rareness

Rareness may be caused by several factors. Undersampling is common in most tropical studies [133]. Rareness does not automatically imply that a species is rare in general. McArdle [147] advised that we should ask when a rare species is really rare or just not there. Under this premise, the actual local abundance of rare species is unknown [99]. Clumped distributions are the rule in nature [148] and can cause rarity [98]. Thus, rarity at a site may be caused by source–sink phenomena or mass effects [30] at both local and regional scales [149,150]. In New Guinea, a species was rare on a particular host, whereas it was more common on other hosts or relatively rare on numerous other host plants, so its aggregate population was high [28]. This has been illustrated for several species of Chrysomelidae [151], Elateridae [69] and Scarabaeidae [152] in the Venezuelan sample. The aggregation of species-rich beetle communities was evident, particularly on flowering [81] and extrafloral nectary-bearing trees [83] in the canopy plot. Additionally, most canopy beetles in the community were associated with their host trees only during their activity, either during the day or at night [69,151–153], causing permanent diel migration. Of the 862 beetle adults collected from the 23 canopy-tree species, 642 species (74.5%) comprising 5175 individuals were collected exclusively on flowers or extrafloral nectaries or on both resources (unpublished data). All of these beetles were restricted in their occurrence to the season and a distinct time of the day; thus, they were not residents of the canopies. Diel and seasonal migration can lead to patterns similar to those of mass effects [133]. Therefore, the beetle migration between trees [69,151,152] is responsible for a large proportion of singletons in tropical rainforest canopies.

Furthermore, rareness may depend on the habitat. Rare species constituted 29% of the carabid beetle community at the highest altitude of 3400 m, and more than 50% were recorded at low altitudes in Peru [154]. Similarly, Floren *et al*. [139] found that the community structure of beetles at an elevation of 1100 m in a Java tropical forest changed from many rare, less-abundant species to communities with a pronounced dominance structure at an elevation of 1700 m. Novotný and Basset [28] were surprised to find that, although more species were caught in the canopy than in the ground layer, this difference was a consequence of the presence of many more rare species in the canopy of a tropical rainforest. On the other hand, Davis *et al*. [155] sampled singletons and doubletons in nocturnal flying beetles with equal distribution across all strata in Sulawesi. The rarity of species at one location may also be caused by the rarity and unpredictability of food resources. While leaf chewers in New Guinea comprised only 8% of unique singletons due to specific adaptations and higher host specificity [28], flower- and extrafloral nectary-visiting beetles face the temporally restricted availability of their food resources. Thus, they track and migrate between appropriate food resources [69,151,152]. This can be augmented by the fact that many rainforest tree species are rare, with average densities of 0.3 to 0.6 trees per species and hectare [156]. In addition, tropical rainforest trees are patchy in their distribution and are probably arrayed in a mosaic or discontinuous pattern [157]. This patchy distribution will be pushed by the fact that there is an overall high diversity of tropical trees, which amounts to 150–200 species of trees per hectare of rainforest, or even more than 300 species per hectare in Amazonia [158]. Moreover, many of these trees flower without regularity and synchrony [55,159,160]; thus, flower resources are not predictable from the perspective of a flower-visiting canopy beetle. In general, the rarity of species in a particular rainforest tree is probably associated with a highly dynamic environment.

## 5. Conclusion

Species richness, community composition and structure of canopy-beetle communities are similar among different tropical rainforests. Tropical rainforest beetle communities are commonly dominated by the herbivorous families Curculionidae and Chrysomelidae. Both dominant families occurred on most tree species in the Venezuelan survey.

In general, the communities comprise a few abundant species, but approximately 50% of singletons. Combining different sampling methods, conducting extended long-term sampling, or collecting taxa on their commonly used food resources can clearly lower the proportion of singletons.

Fluctuating environmental conditions, such as the unpredictability of food resources, together with the overall high biodiversity and low population densities, cause the typical pattern of species abundance distribution in tropical rainforest canopies. A high proportion of the local beetle fauna migrates between food resources. In addition, there is daily migration between resting places and food resources. This migration behaviour is the main reason for the high proportion of singletons within the canopy of tropical rainforests.

## Data Availability

The dataset supporting this article has been uploaded as part of the supplementary material on the Dryad Digital Repository [161].
